# Lipopolysaccharide-Activated Macrophages Suppress Cellular Senescence and Promote Rejuvenation in Human Dermal Fibroblasts

**DOI:** 10.3390/ijms26157061

**Published:** 2025-07-22

**Authors:** Hiroyuki Inagawa, Chie Kohchi, Miyuki Uehiro, Gen-Ichiro Soma

**Affiliations:** 1 Control of Innate Immunity, Collaborative Innovation Partnership, Takamatsu 761-0301, Japan; 2 Macrophi Inc., Takamatsu 761-0301, Japan; 3 Research Institute for Healthy Living, Niigata University of Pharmacy and Applied Life Sciences, Niigata 956-8603, Japan

**Keywords:** senescence, aging, rejuvenation, lipopolysaccharide, macrophage, fibroblast

## Abstract

Tissue-resident macrophages are essential for skin homeostasis. This study investigated whether lipopolysaccharide (LPS)-activated macrophages affect senescence and rejuvenation in human dermal fibroblasts. Human monocytic THP-1 cells were stimulated with *Pantoea agglomerans*–derived LPS (1–1000 ng/mL), and culture supernatants were collected. These were applied to two NB1RGB fibroblast populations: young, actively dividing cells (Young cells) and senescent cells with high population doubling levels and reduced proliferation (Old cells). Senescence markers P16, P21, and Ki-67 were analyzed at gene and protein levels. Conditioned medium from Old cells induced senescence in Young cells, increasing P16 and P21 expression levels. This effect was suppressed by cotreatment with LPS-activated THP-1 supernatant. Old cells treated with the LPS-activated supernatant exhibited decreased P16 and P21 levels as well as increased Ki-67 expression, indicating partial rejuvenation. These effects were not observed following treatment with unstimulated THP-1 supernatants or LPS alone. Overall, these findings suggest that secretory factors from LPS-activated macrophages can suppress cellular senescence and promote human dermal fibroblast rejuvenation, highlighting the potential role of macrophage activation in regulating cellular aging and offering a promising strategy for skin aging intervention.

## 1. Introduction

Normal somatic cells are exposed to various stressors, including inflammation-induced oxidative stress and lysosomal dysfunction, leading to telomere shortening and genomic DNA damage, which ultimately cause irreversible cell cycle arrest and loss of proliferative capacity [1]. When cells reach their replicative limit, they typically enter G1-phase arrest and undergo apoptosis [2,3]. However, pathologically senescent cells often develop resistance to apoptosis and persist in tissues despite growth arrest [4]. These cells secrete proinflammatory factors collectively known as the senescence-associated secretory phenotype (SASP), including cytokines [5], chemokines [6], matrix-degrading enzymes [7], growth factors, and fragmented DNA within exosomes [8]. SASP components contribute to chronic inflammatory diseases and induce senescence-like traits, such as cell cycle arrest and apoptotic resistance, in neighboring healthy cells [9,10].

Promising antiaging strategies include suppressing SASP-induced secondary senescence, efficiently clearing senescent cells, and rejuvenating (i.e., restoring function to) senescent cells [11].

Pathologically senescent cells often evade immune clearance by expressing immune checkpoint molecules, such as PD-L1, which inhibit immune cell function. Therapies using anti-PD-1 antibodies to restore immune surveillance have shown potential for removing senescent cells [12,13,14]. However, few studies have focused on reversing cellular senescence and promoting rejuvenation. This study addresses both the prevention of SASP-induced senescence and the possible rejuvenation of senescent cells.

Specifically, we focus on the antiaging potential of the innate immune system, particularly macrophages. These cells produce reactive oxygen species and proinflammatory cytokines in response to pathogens, contributing to aging-related inflammation [15]. Conversely, tissue-resident macrophages help regulate inflammation and support tissue repair [16], highlighting their dual role in aging [17]. However, their phagocytic capacity reportedly declines with age [18], likely due to age-related microenvironmental changes. Therefore, targeting macrophage activation may offer a novel antiaging strategy that is distinct from the conventional approach of eliminating senescent cells through phagocytosis. Instead, the new method prevents senescence and promotes cellular rejuvenation. Therefore, stimulating macrophages externally may offer a viable antiaging strategy.

We previously demonstrated that oral lipopolysaccharide (LPS) administration safely and effectively enhances macrophage phagocytosis [19,20]. Although LPS is proinflammatory in vitro or when injected, oral delivery does not trigger systemic inflammation but instead activates the phagocytic activity of peritoneal and brain macrophages (microglia). Moreover, orally administered LPS reduces oxidative and inflammatory marker levels, boosts anti-inflammatory and tissue repair marker levels, and ameliorates adverse impacts in aging-related disease models, such as Alzheimer’s disease, diabetic encephalopathy, atherosclerosis (ApoE-deficient mice), and type 2 diabetes (obese KK-Ay mice) models [19,20]. These findings suggest that LPS enhances macrophage reparative and anti-inflammatory functions, improving aging-related pathologies. However, it was unknown whether LPS activation would reverse this decrease in fibroblasts. Therefore, this study investigated whether LPS-activated macrophages can suppress senescence and promote rejuvenation in human dermal fibroblasts.

Accordingly, we hypothesized that LPS-activated tissue-resident macrophages exert antiaging effects not only by clearing senescent cells through phagocytosis but also by suppressing senescence and promoting cellular rejuvenation. To test this, we evaluated the effects of LPS-stimulated macrophage-conditioned media on human dermal fibroblasts. NB1RGB fibroblasts were classified as young or senescent and analyzed for the senescence-associated markers P16, P21, and Ki-67. We found that culture supernatants from senescent fibroblasts induced senescence-like phenotypes in Young cells. However, cotreatment with conditioned media from LPS-stimulated macrophages suppressed this induction. Furthermore, treating senescent fibroblasts with the same media reduced P16 and P21 expression levels while increasing Ki-67 levels, indicating rejuvenation. These findings suggest that targeting macrophage activation may represent a novel strategy for preventing cellular senescence and promoting rejuvenation, distinct from conventional antiaging approaches.

## 2. Results

### 2.1. Suppression of P16 and P21 Expression by LPS-Stimulated Macrophage-Conditioned Media

**Figure 1:**
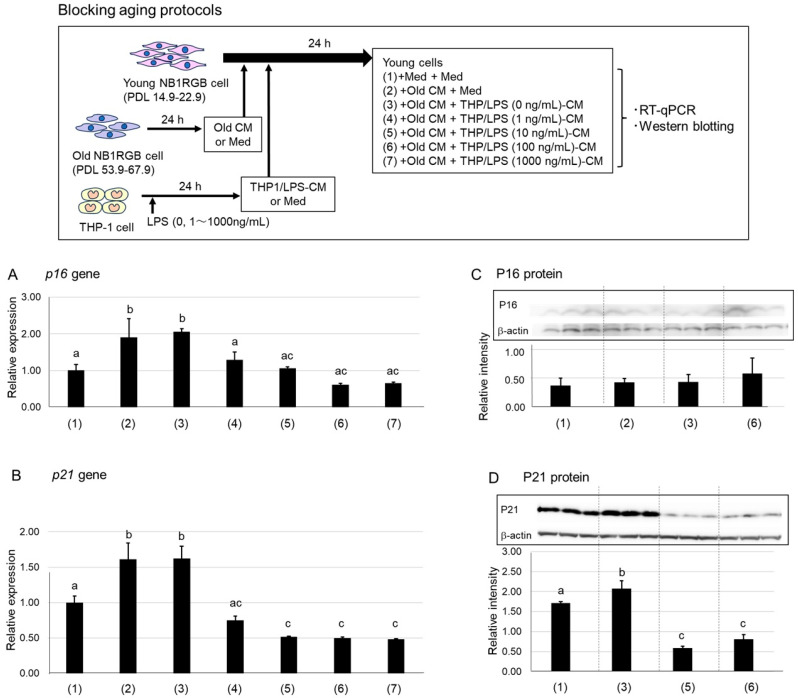
Expression of the *p16* and *p21* genes in Young cells. The schematic protocol (1)–(7) is shown at the top of the figure. The test conditions in figures (**A**–**D**) correspond to the schematic protocol (1)–(7). Young cells were cultured for 24 h after replacing half of the medium with EMEM, Old cell-conditioned medium (Old CM), or THP/LPS-conditioned medium (THP/LPS-CM; 0–1000 ng/mL). (**A**,**B**) Total RNA was extracted, and the relative mRNA expression levels of *p16* (**A**) and *p21* (**B**) were measured by Quantitative real-time PCR (RT-qPCR) using GAPDH as a housekeeping gene. (**C**,**D**) Young cells were treated with EMEM or THP/LPS-CM (0, 10, or 100 ng/mL) for 24 h. Cells were lysed, and 12.5 μg of total protein per lane was separated by SDS-PAGE, followed by western blot analysis using antibodies against P16 (**C**) or P21 (**D**). Representative western blot images are shown in the boxed regions of panels C and D. Band intensities were normalized to β-actin and quantified. Data are presented as mean ± SD (n = 3). Different letters (a–c) indicate statistically significant differences (*p* < 0.05, ANOVA, Tukey–Kramer).

The antiaging potential of macrophages can be assessed from two perspectives: suppressing senescence and promoting rejuvenation. To evaluate senescence suppression, we used a system where SASP factors from senescent cells induce senescence phenotypes in young cells, as previously reported [21]. Overall, THP/LPS-CM remarkably suppressed the expression of senescence markers P16 and P21 in young NB1RGB cells treated with conditioned medium sourced from senescent fibroblasts (Old CM). In contrast, treatment with a conditioned medium from unstimulated THP-1 cells (THP/LPS (0 ng/mL)-CM) had no such effect. Altogether, LPS-activated macrophages secrete factors that counteract SASP-induced senescence in young fibroblasts (Figure 1A,D).

Because human dermal fibroblasts (NB1RGB) and THP-1 macrophage-like cells were cultured in Eagle’s Minimum Essential Medium (EMEM) and RPMI-1640 media, respectively, a 1:1 mixture of these media was used in coculture. Preliminary tests confirmed that this mixture did not alter cell behavior.

We investigated whether conditioned media from LPS-stimulated THP-1 cells (THP/LPS-CM; 0–1000 ng/mL) could suppress the senescence-inducing effects of conditioned media from senescent NB1RG fibroblast cells with high population doubling levels (PDL) and reduced proliferation (Old cells: PDL 53.9–67.9) on young NB1RGB cells (Young cells: PDL 14.9–22.9). When Old CM from senescent Old cells was added to Young cells, mRNA and protein expression levels of P16 and P21, i.e., cyclin-dependent kinase inhibitors and classic cellular senescence markers [22], significantly increased (Figure 1A,B). A similar increase occurred when Old CM was mixed with THP/LPS (0 ng/mL)-CM, confirming that THP-1 supernatants not stimulated with LPS did not block SASP-induced senescence. However, adding THP/LPS (10–1000 ng/mL)-CM significantly lowered *p16* and *p21* expression levels to near-baseline levels in untreated Young cells.

To validate these protein-level effects, we performed western blotting (Figure 1C,D). Although P16 protein expression was too low to detect reliably (Figure 1C), P21 protein expression was significantly elevated in Young cells treated with the Old CM–THP/LPS (0 ng/mL)-CM mixture but significantly reduced in those treated with THP/LPS (10 or 100 ng/mL)-CM (Figure 1D). These results suggest that LPS-activated macrophages secrete factors that neutralize SASP-induced senescence in young fibroblasts.

### 2.2. Rejuvenation of Senescent Cells by THP/LPS-Conditioned Media

**Figure 2:**
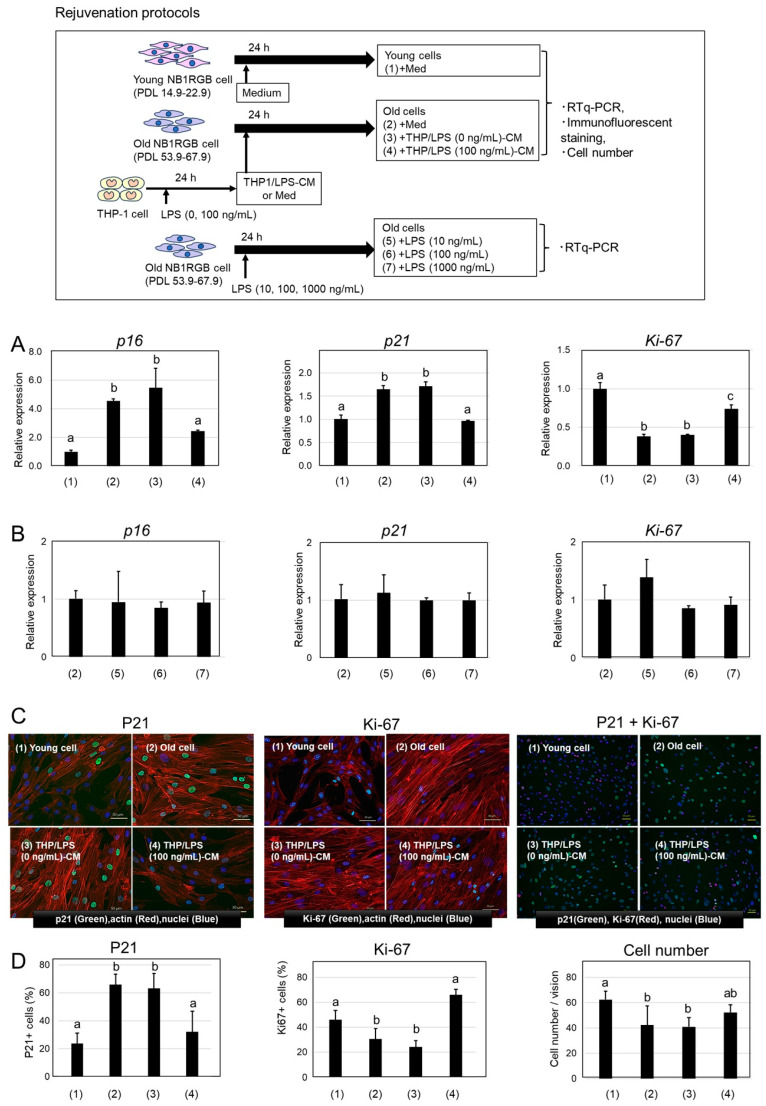
Changes in P16, P21, and Ki-67 expression in senescent NB1RGB cells treated with THP/LPS-CM. The schematic protocol (1)–(7) is shown at the top of the figure. The test conditions in figures (**A**–**D**) correspond to the schematic protocol (1)–(7). (**A**) Young and Old NB1RGB cells were treated with EMEM, THP/LPS (0 ng/mL)-CM, or THP/LPS (100 ng/mL)-CM for 24 h. Relative mRNA expression levels of *p16*, *p21*, and *Ki-67* were analyzed by RT-qPCR and normalized to the levels in Young cells. (**B**) Old cells were treated with LPS (10, 100, or 1000 ng/mL) for 24 h, and the expression levels of *p16*, *p21*, and *Ki-67* were assessed by RT-qPCR. In both (**A**,**B**), bars represent mean ± SD (n = 5). Different letters (a–c) indicate statistically significant differences (*p* < 0.05). (**C**) Young and Old cells were treated with EMEM, THP/LPS (0 ng/mL)-CM, or THP/LPS (100 ng/mL)-CM for 24 h, then immunofluorescence staining was performed using antibodies against P21 and Ki-67 (n = 5). Representative fluorescence images were acquired using a 20× objective lens with 2× digital zoom. In P21 and Ki-67 single-staining panels green = P21 or Ki-67, red = actin, and blue = nuclei. In double-staining panels green = P21, red = Ki-67, and blue = nuclei. Scale bars = 50 μm. (**D**) Based on the immunofluorescence images, the percentages of P21-positive cells, Ki-67-positive cells, and total cell counts per field were quantified. Data are expressed as mean ± SD (n = 5). Different letters (a–c) indicate statistically significant differences (*p* < 0.05, ANOVA, Tukey–Kramer).

Given that THP/LPS-CM suppressed senescence induction in Young cells, we investigated its ability to reverse senescence in Old cells. Specifically, we analyzed mRNA expression levels of *p16*, *p21*, and the proliferation marker *Ki-67* [23] in senescent NB1RGB cells treated with THP/LPS-CM. Notably, treatment of senescent NB1RGB fibroblasts (Old cells) with THP/LPS-conditioned media (THP/LPS-CM) considerably reduced the expression of senescence markers P16 and P21 and increased that of the proliferation marker Ki-67. However, these changes were not observed in cells treated with conditioned media from unstimulated THP-1 cells or when subjected to direct LPS treatment. Thus, factors secreted by LPS-activated macrophages may partially reverse the senescent phenotype and promote rejuvenation (Figure 2A–D).

Old cells exhibited significantly higher *p16* and *p21* levels and lower *Ki-67* levels compared with Young cells, confirming a senescent phenotype (Figure 2A). Conversely, THP/LPS (0 ng/mL)-CM treatment had no such effects. However, treatment with THP/LPS (10–100 ng/mL)-CM significantly reduced *p16* and *p21* expression levels while increasing *Ki-67* levels, suggesting a partial reversal of the senescent phenotype. Importantly, direct treatment of Old cells with LPS (10–1000 ng/mL) alone did not alter *p16*, *p21*, or *Ki-67* expression, indicating that LPS itself does not directly affect senescent fibroblasts (Figure 2B).

To further evaluate protein expression and cellular localization, we conducted immunofluorescent staining for nuclear proteins P21 and Ki-67 (Figure 2C), along with nuclear and actin staining. P16 levels remained too low for analysis; nuclear localization of P21 and Ki-67 was confirmed. In Young cells and THP/LPS (100 ng/mL)-CM–treated Old cells, P21 staining was weak, whereas Ki-67 staining was strong. In contrast, untreated Old cells and those treated with THP/LPS (0 ng/mL)-CM showed strong P21 and weak Ki-67 signals. No distinct morphological changes were observed in the cells.

Quantitative analysis of positive cells and total cell counts per field are shown in Figure 2D. Young cells and THP/LPS (100 ng/mL)-CM–treated Old cells exhibited significantly fewer P21-positive cells and more Ki-67–positive cells compared with untreated and THP/LPS (0 ng/mL)-CM–treated Old cells. Cell numbers per field were also significantly higher in Young and THP/LPS (100 ng/mL)-CM–treated Old cells.

Collectively, these findings demonstrate that THP/LPS-CM treatment modifies the expression of senescence- and proliferation-related markers in senescent fibroblasts, shifting their phenotype toward that of younger cells and indicating a rejuvenating effect.

## 3. Discussion

This study preliminarily assessed the ability of LPS-activated macrophages to prevent and reverse cellular senescence in human dermal fibroblasts (NB1RGB). The senescence markers P16, P21, and Ki-67, previously validated in cellular aging studies, were used to evaluate senescent and rejuvenated phenotypes.

When Old CM was added to Young cells, *p16* and *p21* expression levels significantly increased, confirming SASP-induced senescence (Figure 1). Notably, cotreatment with THP/LPS-CM suppressed this phenotype. This effect was absent with CM from unstimulated THP-1 cells, indicating that LPS activation is necessary to induce protective factors. These results suggest that LPS-stimulated macrophages secrete molecules that counteract SASP-mediated senescence. This finding was unexpected. Previous studies have primarily attributed the antiaging effects of macrophages to the phagocytic clearance of senescent cells. However, the study findings provide the first evidence, stating that macrophages may also exert antisenescent effects via soluble factors and that this is independent of phagocytosis. Overall, these findings suggest a previously unrecognized mechanism by which macrophages may contribute to cellular rejuvenation.

Herein we used THP-1 cells as a representative macrophage-lineage cell model. In vivo, macrophage-lineage cells are primarily composed of tissue-resident macrophages that are highly heterogeneous and adapted to distinct tissue environments. Although various cell lines derived from alveolar, intestinal, and brain macrophages are available, there is currently no universally accepted cell line accurately representing tissue-resident macrophages in general.

Per our experience, undifferentiated THP-1 cells exhibit levels of LPS responsiveness similar to that observed in alveolar, peritoneal, and microglial macrophages. Consequently, we used non-TPA-stimulated THP-1 cells as a model system; additionally, this choice is further supported by previous reports [24,25]. In contrast, TPA-differentiated THP-1 cells show exaggerated responses to LPS but may be unsuitable as a general model of tissue-resident macrophage phenotypes. Therefore, we selected undifferentiated THP-1 cells as a macrophage model capable of displaying tissue-resident-like characteristics, especially concerning innate immune activation by LPS.

Next, we determined whether THP/LPS-CM could reverse the established senescence by treating Old cells directly (Figure 2). Unexpectedly, this treatment resulted in a notable decrease in the gene expression levels of *p16* and *p21*; however, *Ki-67* expression increased. Furthermore, immunofluorescence microscopy revealed a reduction in P21-positive cells and an increase in Ki-67-positive cells. The increased cell number per field suggested a partial restoration of proliferative capacity, indicating rejuvenation. However, we observed no distinct differences in cellular morphology. Notably, direct exposure to LPS alone did not replicate this effect, thereby underscoring the role of macrophage-derived factors in rejuvenation. Overall, only three canonical markers were analyzed; however, these findings offer novel insight into how macrophages are involved in aging.

Herein we clearly observed changes in *p16* gene expression; in contrast, P16 protein levels were too low to detect reliably. Because there is no information on P16 expression in NB1RGB cells, these cells may express P16 only at very low levels. Future work should resolve this question by employing RNAscope to quantify CDKN2A mRNA. Moreover, such future tests should include complementary markers such as LMNB1 and γH2AX.

The specific rejuvenation factors present in the culture supernatant of macrophages stimulated with low-concentration LPS remain unidentified. Reportedly, IL-4, IL-10, and TGF-β—i.e., inflammatory inhibitory type 2 cytokines—are possible candidates [26]. In general, aging is associated with low-grade, chronic inflammation (or inflammaging) that can contribute to many age-related diseases. IL-4 and IL-10 inhibit the production of proinflammatory cytokines such as TNF-α, IL-6, and IL-1β, which are secreted by monocytes/macrophages and other immune cells. These cytokines also suppress NF-κB signaling, a key pathway in age-associated inflammation. Therefore, IL-4 and IL-10 reduce systemic inflammation and help to protect tissues from chronic immune-mediated damage. Moreover, they suppress SASP factors in old macrophages, thereby limiting tissue-damaging effects. Previous studies have also reported downregulation of the expression of P16 and P21, two markers of cellular aging, in some experimental contexts. Taken together, experimental results suggest that IL-4 and IL-10 are important candidate cytokines that may be produced by macrophages in response to LPS stimulation.

Contextually, TGF-β promotes the expression of P16 and P21, thereby contributing to senescence. TGF-β signaling activates the SMAD pathway, potentially inducing the expression of P16 and P21, both of which are involved in cell cycle arrest [27]. Moreover, TGF-β is part of the SASP network and contributes to reinforcing cell cycle exit. In general, LPS stimulates macrophages via Toll-like receptor 4 and promotes their differentiation into M1-type (inflammatory) macrophages. When studied in vitro or in a mouse model, LPS stimulation suppresses TGF-β mRNA expression in macrophages [28]. Additionally, LPS inhibits the activation of the SMAD pathway downstream of TGF-β, thereby leading to a weakened TGF-β response in inflammatory environments. Therefore, the involvement of a mechanism mediated by TGF-β inhibition in LPS-activated macrophage-mediated aging suppression cannot be ruled out. In the future, we plan to investigate whether anti-inflammatory factors suppress SASP production from senescent cells or inhibit the induction of P16 and P21.

Macrophages communicate with their tissue-resident counterparts through soluble factors and chemotaxis. We previously proposed a “macrophage network” concept, where activated macrophages regulate systemic homeostasis beyond localized inflammation and tissue repair [19,20]. For example, oral LPS administration elevates membrane-bound colony-stimulating factor 1 (CSF1) levels in peripheral leukocytes, leading to CSF1 receptor activation on microglia and promoting neuroprotection [29]. This supports the hypothesis that LPS promotes an M2-like (anti-inflammatory) macrophage phenotype, which may help suppress aging and promote tissue renewal.

In this study, we used a simple in vitro model with low volumes of LPS-activated macrophage supernatant to detect indirect effects on fibroblasts [25,30]. Accordingly, we evaluated the preventive and restorative properties of macrophage-derived factors in cellular senescence. Tissue-resident macrophages, essential for immune surveillance, tissue repair, and regeneration, differ by tissue origin and activation state. For instance, in bone remodeling, osteoclasts (specialized macrophages) interact with osteoblasts to maintain skeletal homeostasis [31]. Macrophages are broadly classified into proinflammatory M1 and anti-inflammatory/regenerative M2 subtypes. In the skin, M1 macrophages accumulate with age, whereas M2 macrophages are more prevalent in younger tissue [17]. Moreover, aging diminishes the phagocytic and anti-inflammatory functions of cutaneous macrophages [32], contributing to disease states, such as chronic kidney disease [33]. These observations underscore the importance of maintaining macrophage balance for tissue homeostasis and youthfulness. Topical application of *Pantoea agglomerans*–derived LPS has been shown to reverse age-related epidermal thinning in mice [34], aligning with our finding that LPS-activated macrophages can rejuvenate senescent human fibroblasts.

Although LPS is commonly associated with proaging effects due to its capacity to trigger ROS and proinflammatory cytokine production [35], these effects typically arise from systemic injection in disease models [36]. In contrast, low-level LPS from symbiotic gram-negative bacteria on mucosal surfaces (e.g., the skin, gut, and airways) may produce anti-inflammatory and reparative responses. Oral or topical low-dose LPS has shown benefits in models of diabetes, atherosclerosis, dementia, and allergic dermatitis [19,20]. In vitro models simulating oral LPS exposure also support the potential of low-dose LPS to activate macrophages [25].

The *P. agglomerans*–derived LPS used in this study has low inflammatory potential when ingested or applied dermally and has a long-standing safety record [19,20,37]. This bacterium, found in the rhizosphere of crops such as wheat and rice, is recognized as a plant growth-promoting symbiont [38] and has likely been ingested by humans over generations through natural dietary exposure [37].

This study presents preliminary in vitro evidence that LPS-activated macrophages may suppress senescence and promote rejuvenation in human dermal fibroblasts. However, several limitations should be acknowledged. First, the findings are limited to a cell culture model, and it remains unclear whether similar effects occur in vivo. In particular, the diffusion and functional action of macrophage-derived factors within tissue environments were not evaluated. Second, assessment of cellular senescence was based on only three markers—P16, P21, and Ki-67—which may not fully assess the multifaceted nature of senescence. Future studies should consider including additional markers such as β-galactosidase staining, evaluation of LMNB1 reduction, and detection of DNA damage markers such as γH2AX. Third, the specific soluble factors secreted by LPS-stimulated macrophages responsible for the observed effects were not identified. Further studies are needed to elucidate the underlying mechanisms. Additionally, the THP-1 cells used in this study are a monocytic cell line and may not fully reflect the behavior of tissue-resident macrophages in human skin. To validate this hypothesis, future research should explore in vivo models and macrophage cocultures to elucidate the mechanisms and therapeutic potential of macrophage-based antiaging strategies [39].

In summary, LPS-activated macrophages appear to play dual roles: preventing senescence onset and inducing phenotypic rejuvenation in senescent cells. To our knowledge, this is the first report implicating macrophage-mediated rejuvenation via LPS stimulation. Similar mechanisms have been proposed in diabetic dementia prevention and treatment [29]. We hypothesize that the macrophage network functions as a dynamic biological control system mediating LPS-induced protective effects [19,20]. The antisenescence and rejuvenating effects observed in this study may represent one facet of this network.

## 4. Materials and Methods

### 4.1. Cell Culture

The human dermal fibroblast cell line NB1RGB, with various PDLs, was obtained from the RIKEN BioResource Center (Ibaraki, Japan). Cells with low PDLs (14.9–22.9), exhibiting high proliferative capacity, were designated as Young cells, whereas those with high PDLs (53.9–67.9), displaying reduced proliferation and elevated *p16* and *p21* expression, were defined as Old cells. CM from Old cells induced increased *p16* and *p21* expression in Young cells, confirming their senescent phenotype.

All NB1RGB cells were cultured in EMEM (FUJIFILM Wako Pure Chemical Corporation, Osaka, Japan) supplemented with 10% fetal bovine serum (FBS; Hyclone, Funakoshi Co., Tokyo, Japan) at 37 °C in a humidified atmosphere with 5% CO_2_. For Young cells, the culture medium was replaced with fresh EMEM containing 10% FBS 3 days before experiments began. Old CM was prepared by culturing Old cells in EMEM with 10% FBS for 7 days, followed by filtration through a 0.2 μm membrane. To assess the direct effects of lipopolysaccharide (LPS), Old cells were treated with *Pantoea agglomerans*–derived LPS (Macrophi Inc., via Funakoshi Co., Tokyo, Japan) at final concentrations of 1, 10, 100, or 1000 ng/mL for 24 h.

### 4.2. CM Preparation

The human monocytic cell line THP-1 (ATCC TIB-202; Sumisho Pharma International Inc., Tokyo, Japan) was cultured in RPMI-1640 medium (FUJIFILM Wako Pure Chemical Corporation, Osaka, Japan) supplemented with 10% FBS. Cells were stimulated with LPS at final concentrations of 1, 10, 100, or 1000 ng/mL and incubated for 24 h. The resulting supernatants were collected and designated as THP/LPS-CM. Supernatants from unstimulated THP-1 cells served as controls (THP-CM).

### 4.3. Stimulation of NB1RGB Cells with CM

#### 4.3.1. Inhibition of Senescence Induction

Young cells (3 × 10^5^ cells/3 mL/well) were seeded in 6-well plates and cultured for 24 h (n = 3). The medium was then replaced with one of the following:

(1) EMEM alone
(2) Old CM alone,
(3) A 1:1 mixture of Old CM and THP/LPS (0 ng/mL)-CM,
(4) A 1:1 mixture of Old CM and THP/LPS (1 ng/mL)-CM,
(5) A 1:1 mixture of Old CM and THP/LPS (10 ng/mL)-CM,
(6) A 1:1 mixture of Old CM and THP/LPS (100 ng/mL)-CM,
(7) A 1:1 mixture of Old CM and THP/LPS (1000 ng/mL)-CM.

All media were supplemented with 10% FBS, and cells were incubated for an additional 24 h. Figure 1 shows a schematic illustrating this experimental design.

#### 4.3.2. Rejuvenation of Senescent Cells

Old or Young cells were seeded in 6-well plates at 3 × 10^5^ cells/3 mL and cultured for 24 h (n = 5). The medium was then replaced with one of the following:

(1) EMEM alone,
(2) EMEM medium alone,
(3) THP/LPS (0 ng/mL)-CM,
(4) THP/LPS (100 ng/mL)-CM,
(5) LPS (10 ng/mL) alone,
(6) LPS (100 ng/mL) alone,
(7) LPS (1000 ng/mL) alone.

(1) are Young cells, and (2)–(7) are Old cells. All treatments were carried out in EMEM supplemented with 10% FBS. Cells were incubated for 24 h. A graphical schematic illustrating this experimental design is shown in Figure 2.

### 4.4. RNA Extraction and RT-qPCR

Total RNA was extracted using the FastGene™ RNA Basic Kit (Nippon Genetics Co., Tokyo, Japan) following the manufacturer’s instructions. RNA concentration was measured via absorbance at 260 nm. cDNA was synthesized using ReverTra Ace™ qPCR RT Master Mix with gDNA remover (TOYOBO Co., Osaka, Japan). Primers for *p16*, *p21*, *Ki-67*, and GAPDH were designed and synthesized by Fasmac Co. (Kanagawa, Japan) (Table 1).

RT-qPCR was performed using the THUNDERBIRD™ SYBR™ qPCR Mix (TOYOBO Co., Osaka, Japan) under the following cycling conditions: 95 °C for 1 min, followed by 45 cycles of 95 °C for 15 s and 60 °C for 1 min. A final dissociation step was performed at 95 °C for 1 min, 55 °C for 30 s, and 95 °C for 30 s. GAPDH served as the internal control. Results are expressed as fold-changes relative to untreated Young cells.

### 4.5. Western Blotting

After CM treatment, NB1RGB cells were washed with PBS and lysed in RIPA buffer (FUJIFILM Wako Pure Chemical Corporation, Osaka, Japan) containing a protease inhibitor cocktail (Complete™, Merck KGaA, Darmstadt, Germany). Lysates were centrifuged at 14,500 rpm and 4 °C for 20 min, and the supernatants were collected. Protein concentrations were determined using the Pierce™ BCA Protein Assay Kit (Thermo Fisher Scientific, Waltham, MA, USA) and adjusted to 12.5 μg per lane.

Proteins were resolved via 12.5% SDS-PAGE at 30 mA for 90 min and transferred onto PVDF membranes at 300 mA for 1 h. Membranes were then blocked with PBS containing 0.1% Tween-20 and 5% skim milk for 1 h, followed by incubation overnight at 4 °C with primary antibodies against P21 Waf1/Cip1 (1:1000) and β-actin (1:2000; both from Cell Signaling Technology, Danvers, MA, USA). After washing, membranes were incubated with HRP-conjugated antirabbit or antimouse IgG secondary antibodies (1:1000) for 1 h at room temperature. Bands were visualized using WesternBright ECL HRP substrate (Advansta Inc., San Jose, CA, USA) and imaged using the Amersham™ Imager 680 (Global Life Sci Tech Japan, Tokyo, Japan). Band intensities were quantified and normalized against β-actin.

### 4.6. Immunofluorescence Staining

Following CM treatment, cells were fixed with 4% paraformaldehyde (Nacalai Tesque, Kyoto, Japan) for 15 min at room temperature, washed three times with PBS, and blocked for 60 min. They were then incubated overnight at 4 °C with primary antibodies against P21 (1:500) or Ki-67 (1:10,000; Cell Signaling Technology).

After washing, cells were incubated for 2 h at room temperature in the dark with Alexa Fluor^®^ 488–conjugated secondary antibodies (antirabbit or antimouse IgG, 1:1000; Cell Signaling Technology). Nuclei were stained with NucBlue™ Live ReadyProbes™ Reagent (Hoechst 33342; Thermo Fisher), and actin was stained using Phalloidin-iFluor™ 555 Conjugate (Cayman Chemical, Ann Arbor, Michigan, MI, USA). Fluorescence images were captured using a BZ-X810 microscope (20× objective lens, 2× zoom; Keyence Corp., Osaka, Japan). Percentages of P21- and Ki-67-positive cells were quantified using BZ-H4A, BZ-H4C, and BZ-H4CM analysis software version 1.1.2.4 (Keyence, Osaka, Japan).

### 4.7. Statistical Analysis

RT-qPCR, western blotting, and immunofluorescence data were statistically analyzed using BellCurve for Excel (ver. 4.04; Social Survey Research Information Co., Ltd., Tokyo, Japan). One-way analysis of variance (ANOVA) followed by the Tukey–Kramer multiple comparison test was also applied for analysis, with *p* < 0.05 considered statistically significant.

## 5. Conclusions

This study demonstrates that macrophages activated by LPS can suppress senescence and promote rejuvenation in human dermal fibroblasts. These findings challenge the prevailing notion that LPS primarily promotes aging and inflammation. The observed antisenescent effects of LPS-activated macrophages highlight a novel and unexpected mechanism, offering novel avenues for the development of therapeutic strategies targeting cellular aging. Future studies should identify the specific secreted factors that are responsible for the observed effects and should then validate these findings using in vivo models of skin aging.

## Figures and Tables

**Table 1:** Primer sequences used for RT-qPCR.

Gene		Sequences	Gene Bank ID
*p16*	F	GAG CAG CAT GGA GCC TTC	NM_000077
	R	CCT CCG ACC GTA ACT ATT CG	
*p21*	F	GGA CAG CAG AGG AAG AC	NM_000389
	R	GGC GTT TGG AGT GGT AGA AA	
*Ki-67*	F	TCC CGC CTG TTT TCT TTC TGA C	NM_002417
	R	CTC TCC AAG GAT GAT GAT GCT TTA C	
GAPDH	F	CGA GAT CCC TCC AAA ATC AA	NM_002046
	R	GGT GCT AAG CAG TTG GTG GT	

## Data Availability

The raw data supporting the conclusions of this article will be made available by the authors on request (ciitra@shizenmeneki.org).

## Abbreviations

The following abbreviations are used in this manuscript:

DNA	deoxyribonucleic acid
CM	conditioned medium
PCR	polymerase chain reaction
PD-L1	programmed death ligand 1
RNA	ribonucleic acid
